# Effects of Lysophosphatidylcholine on Intestinal Health of Turbot Fed High-Lipid Diets

**DOI:** 10.3390/nu14204398

**Published:** 2022-10-20

**Authors:** Sihui Li, Xing Luo, Zhangbin Liao, Mengqing Liang, Houguo Xu, Kangsen Mai, Yanjiao Zhang

**Affiliations:** 1 The Key Laboratory of Aquaculture Nutrition and Feed (Ministry of Agriculture), The Key Laboratory of Mariculture (Ministry of Education), Ocean University of China, 5 Yushan Road, Qingdao 266003, China; 2 Yellow Sea Fisheries Research Institute, Chinese Academy of Fishery Sciences, 106 Nanjing Road, Qingdao 266071, China; 3 Qingdao National Laboratory for Marine Science and Technology, 1 Wenhai Road, Qingdao 266237, China

**Keywords:** lysophospholipid, intestinal histological analysis, Toll-like receptor, pro-inflammatory cytokines, apoptosis, intestinal mucosal barrier, intestinal microbiota, turbot

## Abstract

An 8-week feeding trial was conducted, where turbot were fed four experimental diets, containing different LPC levels (0%, 0.1%, 0.25%, and 0.5%, named LPC0, LPC0.1, LPC0.25, and LPC0.5, respectively). The intestinal morphology results showed that there were no widened lamina propria and mixed inflammatory cells in the LPC-supplemented groups. Dietary LPC remarkably decreased the expression of TLRs (TLR3, TLR8, TLR9, and TLR22), MyD88, and signaling molecules (NF-κB, JNK, and AP-1). Similarly, diets with LPC supplementation markedly depressed the gene expression of NF-κB and JNK signaling pathway downstream genes (TNF-α, IL-1β, Bax, Caspase9, and Caspase-3). Furthermore, dietary LPC modified the intestinal microbial profiles, increasing the relative abundance of short-chain fatty acids-producers, lactic acid bacteria, and digestive enzyme-producing bacteria. Predictive functions of intestinal microbiota showed that turbot fed LPC diets had a relatively higher abundance of functions, such as lipid metabolism and immune system, but a lower abundance of functions, such as metabolic diseases and immune system diseases. The activities of intestinal acid phosphatase and alkaline phosphatase were also increased by dietary LPC. In conclusion, LPC supplementation could regulate the intestinal mucosal barrier via the TLR signaling pathway and alter the intestinal microbiota profile of turbot fed high-lipid diets.

## 1. Introduction

It is well-known that the intestine is not only the organ for lipid digestion and absorption, but also a vital barrier to the host’s health. Inflammatory cytokines, such as TNF-α and IL-1β, secreted by immune cells, participate in the maintenance of the intestinal immune barrier [1]. Meanwhile, the proliferation of intestinal epithelial cells is essential for the intestinal physical barrier [2]. The intestinal microbiota constitutes a complex community and interacts with the host to modulate essential biological processes of health maintenance [3]. There were plenty of studies reporting that the intestinal microbiota has a necessary effect on the regulation of fish health [4,5,6]. Additionally, the intestinal brush border enzymes were also involved in protecting the intestinal barrier, and some of them were even associated with the maintenance of intestinal microbial homeostasis [7,8].

Toll-like receptors (TLR) are the crucial pattern recognition receptors, recognizing non-self and danger signals of the immune system and further inducing innate immunity [9,10]. TLR-mediated signals are mainly transduced through the adaptor protein, myeloid differentiation primary response gene 88 (MyD88), while only signals triggered by TLR3 directly initiate transcription factor interferon regulatory factors (IRF3). MyD88 sequentially results in the activation of the downstream regulator molecules, such as nuclear factor κB (NF-κB) and mitogen-activated protein kinases (MAPKs), which could further enhance the expression of pro-inflammatory cytokines and pro-apoptotic factors [11,12]. In modern aquaculture, in order to spare dietary protein, the use of high-lipid diets is becoming more and more popular. However, a variety of problems resulting from high-lipid diets have occurred, including damage of fish intestines [13,14,15]. Accumulating evidence indicates that intestinal inflammation induced by high levels of dietary lipid is associated with TLR-signal transduction in both mammals and fish [16,17,18]. Therefore, the inhibition of the TLR signaling pathway might be essential for the mitigation of intestinal damage caused by high-lipid diets.

As an emulsifier-like feed additive, lysophosphatidylcholine (LPC) has been widely used in terrestrial animals [19,20,21,22]. A recent study has demonstrated that the promotion of broilers intestinal morphology by dietary LPC was closely related to TLR signaling pathways [20]. Furthermore, it has been reported that dietary LPC was effective in regulating the intestinal microbiota of terrestrial animals, such as broilers, sows, and piglets [19,21,22]. In fish, to date, some studies in hybrid tilapia (*Oreochromis aureus* *♂* *× Oreochromis niloticus* *♀*), channel catfish (*Ictalurus punctatus*), turbot (*Scophthalmus maximus* L.), rainbow trout (*Oncorhynchus mykiss*), and the large yellow croaker (*Larimichthys crocea*) have investigated the effects of dietary LPC on fish growth and physiology [23,24,25,26,27,28]. However, few studies have investigated the effects of dietary LPC on fish intestinal health, including the regulation of intestinal mucosal barrier and intestinal microbiota. Previous studies about emulsifiers focused on their functions in lipid digestion. Comprehensive evaluation of the effects of emulsifiers on the intestinal health status of fish are beneficial to better management of emulsifiers in fish feeds.

Turbot (*Scophthalmus maximus* L.) is an extensively cultured marine carnivorous fish all over the world. The lipid requirement for turbot is around 10% [29]. A previous study in turbot has elucidated that high levels of dietary lipid resulted in intestinal damage [14]. The present study was mainly aimed at exploring the effects of dietary LPC on the intestinal health of turbot fed high-lipid diets.

## 2. Materials and Methods

### 2.1. Ethics Statement

All experimental protocols were approved by the Animal Care and Use Committee of Yellow Sea Fisheries Research Institute (recorded case No.: IACUC202003154258).

### 2.2. Experimental Diets

The lysophosphatidylcholine (LPC) product (powder; soy-derived; phosphatidylcholine purity, 98%; available LPC concentration, 5%) used in the present study was supplied by Weifang Kenon Biological Technology Co., Ltd. (Weifang, China). A previous study in turbot has reported that 0.1–0.55% dietary LPC enhanced the fish growth of turbot fed diets with normal lipid levels [24]. To explore whether these doses of LPC supplementations could also be effective in turbot fed high-lipid diets, four isonitrogenous and isolipidic experimental diets were formulated, containing different levels of LPC, namely, 0%, 0.1%, 0.25%, and 0.5% (Table 1). The crude protein content in the control diet was approximately 44% and the crude lipid content was 15%. The four experimental diets were designated as LPC0 (Control), LPC0.1, LPC0.25, and LPC0.5, respectively. According to the standard procedures, all diets were pelleted with a single-screw pelleting machine, dried for 12 h in a ventilated oven at 55 °C, and stored at −20 °C until used [30].

### 2.3. Feeding Trial

Healthy juvenile turbots (initial body weight, appr. 8 g) used in the present study were purchased from Huanghai Aquaculture Co. Ltd. (Haiyang, China). After acclimation to the experimental conditions, similar-size fish were distributed into 12 polyethylene tanks (200 L, 30 fish per tank). Each diet was randomly assigned to triplicate tanks. Fish were fed to apparent satiation twice daily (7:30 and 17:30) for 8 weeks. All 12 tanks were supplied with flow-through deep-well seawater in both the acclimation period and the feeding period. During the feeding trial, the water temperature ranged from 16.2 to 16.6 °C; salinity, 27–29; pH, 7.4–7.9; and dissolved oxygen, 7.5–8.1 mg/L.

### 2.4. Sample Collection

Experimental fish were fasted for 24 h, and then anesthetized with eugenol (1:10,000) (purity, 99%, Shanghai Reagent Corp, Shanghai, China) before sampling. All fish were counted and weighed. After that, for the analysis of the intestine somatic index and intestinal length index, two fish were randomly selected from each tank. For the analysis of the intestinal histology, the distal intestines of three fish per tank were randomly collected and fixed with Bouin’s (G-clone, Beijing, China) fixative solution, and then transferred into 70% ethanol after 24 h. For the analysis of gene expression and enzyme activity, the distal intestines of three fish per tank were randomly collected and transferred to 1.8 mL sterile RNase-free cryogenic tubes (Nunc, New York, NY, USA), frozen in liquid nitrogen, and stored at −80 °C. For the intestinal microbiological analysis, the distal intestines of three fish per tank were randomly collected and the entire sampling process was performed under sterile conditions. All scissors, tweezers and rubber blades were disinfected with 70% alcohol. After the intestinal luminal contents were removed, the distal intestinal mucosa was carefully scraped and then the sample was transferred into 2-mL sterile tubes (Corning, NY, USA), frozen with liquid nitrogen, and stored at −80 °C.

### 2.5. Intestinal Length Index and Intestinal Somatic Index

When the samples were collected, the length and weight of the whole fish and their intestines were measured. The following variables were calculated:

Intestinal length index (ILI, %) = intestine length (cm)/fish body length (cm) × 100

Intestinal somatic index (ISI, %) = intestine weight (g)/fish body weight (g) × 100

### 2.6. Intestinal Histology

Preserved tissue segments were routinely dehydrated in ethanol, equilibrated in xylene and embedded in paraffin. Sections of approximately 5 μm from the middle part of distal intestines were cut with microtome (Leica RM2235, Leica Biosystems Co. Ltd. Wetzlar, Germany) and stained with haematoxylin and eosin (H&E). To observe the changes in intestinal histology following the commonly used criteria, the examination of slides was conducted with a light microscope (DP72, Olympus, Japan) equipped with a camera (E600, Nikon, Tokyo, Japan) and CellSens Standard Software (Olympus) [31]. The height of mucosal folds (hMF), muscularis mucosa (hMM), microvilli (hMV), and perimeter ratio (PR) was measured and calculated using the Image Pro Plus^®^ software (Media Cybernetics, Silver Spring, MD, USA). The PR equals the internal perimeter (IP) of the intestine lumen (villi and mucosal folding length) relative to the external perimeter (EP) of the intestine (PR = IP/EP) [32].

### 2.7. Intestinal Enzyme Activities

The distal intestines were thawed, weighed, and homogenized in a cold saline solution. They were then centrifuged at 2500 rpm for 10 min at 4 °C. The supernatant was collected for the analysis of enzyme activity. The total protein concentration in the supernatant was determined with a BCA protein assay kit (#P0010, Beyotime Biotechnology, Shanghai, China). Acid phosphatase (ACP; A060-2-1) and alkaline phosphatase (AKP; A059-2-2) were determined by commercially available kits (Nanjing Jiancheng Bioengineering, Nanjing, China).

### 2.8. RNA Extraction and Real-Time PCR

The total RNA of the distal intestines was isolated using MolPure^®^ Cell/Tissue Total RNA Kit (19221ES50; Yeasen Biotechnology Co., Ltd., Shanghai, China). After the integrity of extracted RNA was assayed by electrophoresis on a 1.2% (*w*/*v*) agarose gel, the concentration and quality of extracted RNA were assessed with NanoDrop ND-2000 Spectrophotometer (Thermo Scientific, Waltham, MA, USA). The cDNA was generated by reversely transcribing 2000 ng RNA in 20 μL reactions using Hifair^®^ III 1st Strand cDNA Synthesis SuperMix for qPCR (11141ES10; Yeasen Biotechnology Co., Ltd., Shanghai, China). The real-time PCR was performed in a total of 20 μL volume containing 1 μL cDNA template (≤50 ng); 0.4 μL Forward primer (10 mM); 0.4 μL Reverse primer (10 mM); 8.2 μL DEPC-treated water (Sangon Biotech, Shanghai, China); and 10 μL SYBR^®^ Green Premix Pro Taq HS qPCR Kit (AG11701, Accurate Biotechnology (Hunan) Co., Ltd., Changsha, China). The qRT-PCR program was as follows: 30 s at 95 °C, followed by 40 cycles of 5 s at 95 °C and 30 s at 60 °C.

The primers for target genes and housekeeping genes were designed in NCBI and synthesized by Sangon Biotech (Shanghai) Co., Ltd. (Table S1). The amplification efficiency for all primers, which was estimated with standard curves based on a 4-step 5-fold dilution series of target template, was within 95~105%, and the coefficients of linear regression (R^2^) were >0.99. The gene expression levels were calculated by the 2^−ΔΔCT^ method [33].

### 2.9. Intestinal Microbiota DNA Extraction, Sequencing and Analysis

Genomic DNA of the distal intestines was extracted by Novogene Genomics Technology Co., Ltd. (Beijing, China). To characterize bacterial community structures and compositions, the V4 region of 16S rRNA gene was amplified with the primer 515F/806R [34]. The Illumina NovaSeq platform was used for sequencing.

After the barcode and primer sequence were cut off, paired-end reads were merged using FLASH software [35]. The high-quality clean tags were obtained according to QIIME [35]. The effective tags were finally obtained using UCHIME algorithm and then were assigned to the same operational taxonomic units (OTUs) by UPARSE based on 97% sequence similarity [36,37]. Representative sequence for each OTU was screened for further annotation using the Silva Database (v132) based on the RDP classifier. Taxonomic assignment was performed using the RDP classifier based on the reference database (Greengenes database) [38]. The alpha diversity analysis (observed species, Chao1, ACE, Simpson index, and Shannon index) and beta diversity on unweighted UniFrac for principal coordinates analysis (PCoA), non-metric multidimensional scaling (NMDS), and unweighted pair group method with arithmetic mean (UPGMA) clustering were calculated with QIIME and displayed with R software (v 3.6.2) [34]. An analysis of molecular variance (AMOVA) test was employed to assess the difference of microbial composition between groups using the adegenet package in R software (version 4.1.0). MetaStat analysis was used to evaluate the bacterial taxa differentially presented between groups of taxa [39]. To explore the metabolic pathway of the microbial communities found in all groups, a bioinformatics tool, Phylogenetic Investigation of Communities by Reconstruction of Unobserved States (PICRUSt), was used to annotate the Kyoto encyclopedia of genes and genomes (KEGG) pathway [40]. The metagenome prediction was further classified into KEGG Orthologs (KOs) at different pathway levels [40].

### 2.10. Statistical Analysis

The statistical software SPSS 23.0 for Windows (IBM SPSS Corporation, Chicago, IL, USA) was used for the data analysis. The results were analyzed with a one-way analysis of variance (ANOVA), and Tukey’s multiple range test was used for the multiple comparisons between group means [41]. Differences were regarded as significant when *p* < 0.05.

## 3. Results

### 3.1. Intestinal Length Index and Intestinal Somatic Index

There was no significant difference in intestinal length index (ILI) and intestinal somatic index (ISI) among all groups (*p* > 0.05) (Figure S1).

### 3.2. Intestinal Histology

**Figure 1:**
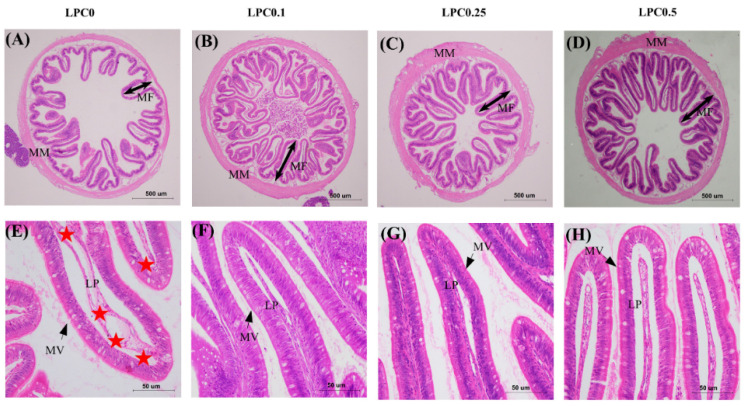
Histological sections of the distal intestine in four experimental groups. Scale bar = 500 μm for (**A**–**D**), and scale bar = 50 μm for (**E**–**H**). Staining: H & E. Red stars showed the infiltration of mixed leucocytes in the lamina propria (LP). MF mucosal folds. MM muscularis mucosa. MV microvilli (black arrows).

**Figure 2:**
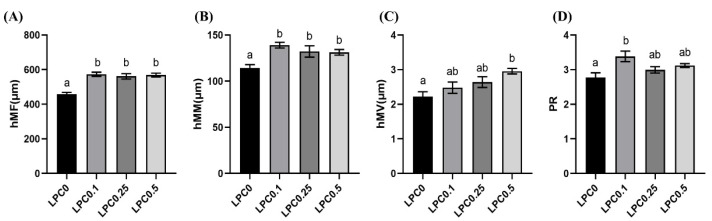
Intestinal histology parameters. (**A**) The height of mucosal folds (hMF); (**B**) The height of muscularis mucosa (hMM); (**C**) The height of microvilli (hMV); (**D**) perimeter ratio (PR). Results were expressed as means ± S.E., and different superscript letters indicate significant differences (*p* < 0.05).

As shown in Figure 1E,F, the infiltration of mixed leucocytes, widened lamina propria, as well as disordered arrangement microvilli were observed in the distal intestine in the LPC0 group, but these symptoms were not observed in the LPC-supplemented groups. Compared with the LPC0 group, the height of mucosal folds (hMF) and muscularis mucosa (hMM) were visibly increased in the three LPC-supplemented groups, while the height of microvilli (hMV) and perimeter ratio (PR) were significantly increased only in the LPC0.1 and LPC0.5 group, respectively (*p* < 0.05) (Figure 2).

### 3.3. Intestinal Enzyme Activities

**Figure 3:**
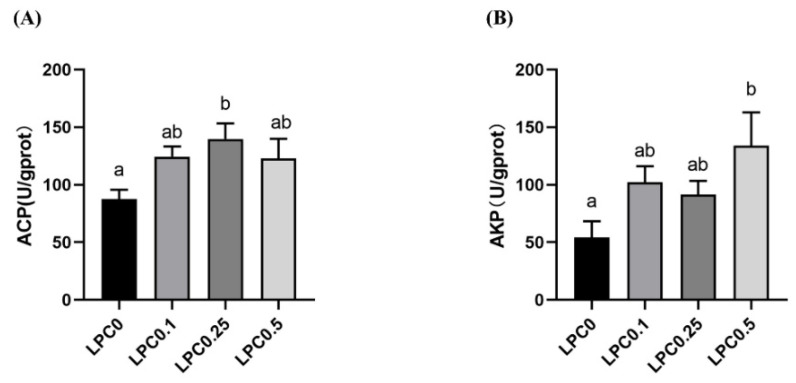
The intestinal enzyme activities of experimental turbot. (**A**) Alkaline Phosphatase (AKP); (**B**) Acid phosphatase (ACP). Results were expressed as means ± S.E., and different superscript letters indicate significant differences (*p* < 0.05).

The activities of acid phosphatase (ACP) and alkaline phosphatase (AKP) were increased by dietary LPC, and the highest activities of ACP and AKP were observed in the LPC0.25 and LPC0.5 group, respectively (*p* < 0.05) (Figure 3).

### 3.4. Gene Expression

#### 3.4.1. Gene Expression of TLR Signaling Pathway

**Figure 4:**
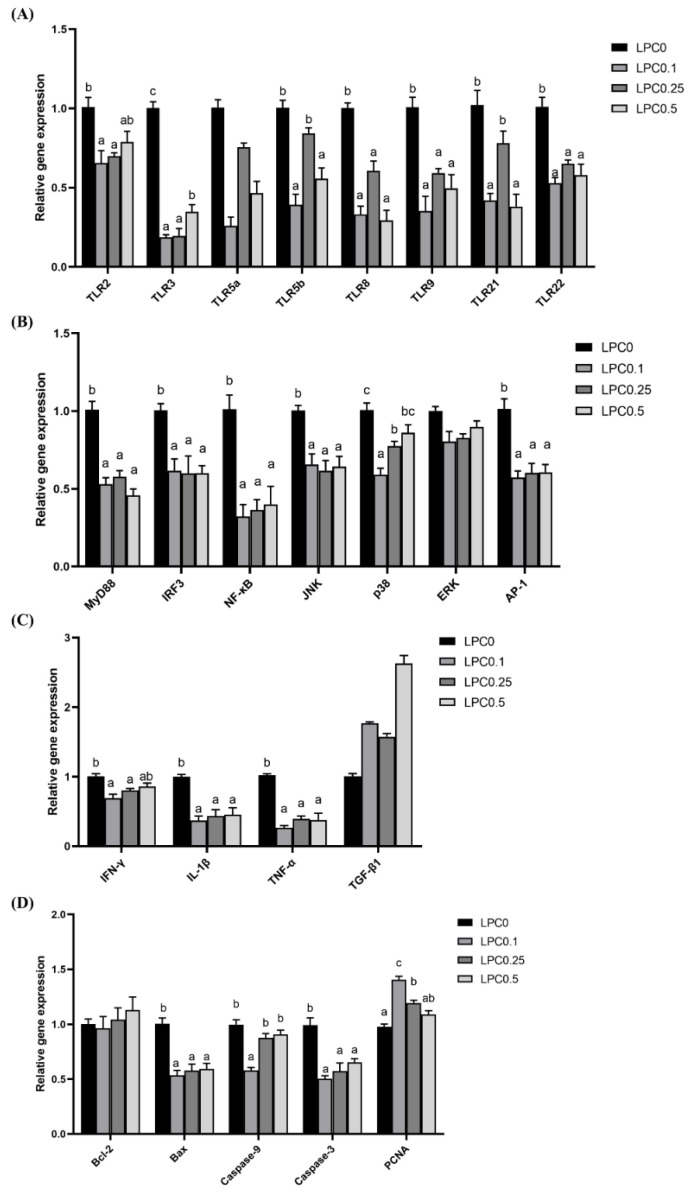
Gene expression of TLR signaling pathway and intestinal mucosal barrier-related factors in the distal intestines (*n* = 6). (**A**,**B**) Gene expression of Toll-like receptors (TLRs), adaptor proteins, and downstream regulator molecules; (**C**) Gene expression of inflammatory cytokines; (**D**) Gene expression of epithelial cell apoptosis and proliferation. Results were expressed as means ± S.E., and different superscript letters indicate significant differences (*p* < 0.05).

Diets with 0.1–0.5% LPC supplementation significantly decreased the genes expression of TLR3, TLR8, TLR9, and TLR22 (*p* < 0.05) (Figure 4A).

As shown in Figure 4B, LPC supplementation significantly reduced the gene expression of the adaptor protein (MyD88) and the downstream regulator molecules of the TLR signaling pathway, such as IRF3, NF-κB, JNK, and AP-1 (*p* < 0.05).

#### 3.4.2. Gene Expression of Inflammatory Cytokines

Diets with LPC supplementation significantly downregulated the gene expression of IL-1β and TNF-α, while dietary LPC at 0.1% and 0.25% significantly down-regulated the IFN-γ expression (*p* < 0.05). No significant difference was observed in the expression of TGF-β1 among all groups (*p* > 0.05) (Figure 4C).

#### 3.4.3. Gene Expression of Epithelial Cell Apoptosis and Proliferation

Dietary LPC evidently downregulated the gene expression of pro-apoptotic factors including Bax, Caspase-9, and Caspase-3 (*p* < 0.05). Compared to LPC0 groups, the expression of PCNA was remarkably upregulated by dietary LPC with 0.1% and 0.25% supplementation and the highest value was observed in the LPC0.1 group (*p* > 0.05) (Figure 4D).

### 3.5. Intestinal Microbiota

**Figure 5:**
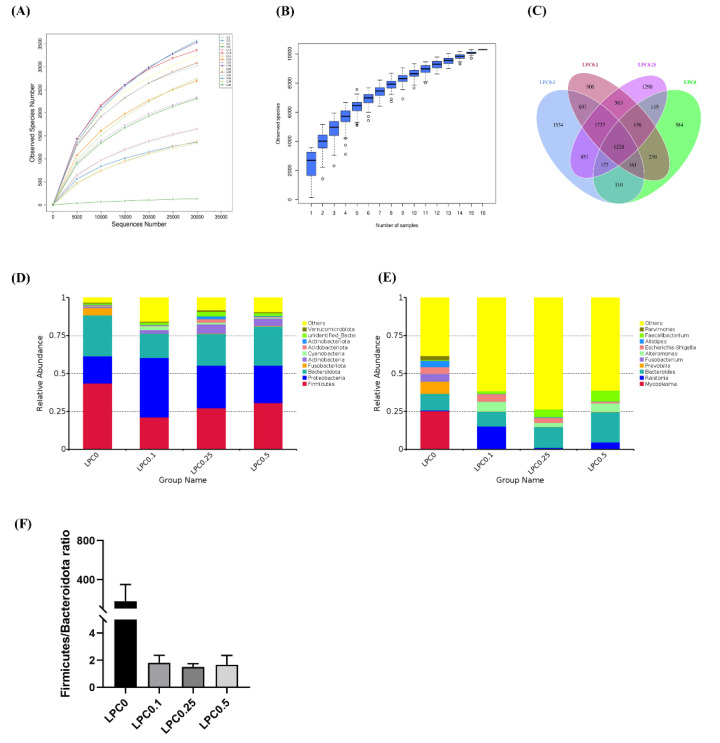
Taxonomy classification of reads at different levels (*n* = 4). (**A**) Rarefaction curve; (**B**) Species accumulation boxplot; (**C**) Venn plot; (**D**,**E**) Bacteria taxonomic profiling at the phylum and genus level; (**F**) The Firmicutes/Bacteroides ratio.

After assembly, quality control, and splicing, a total of 1,008,526 high-quality effective tags were obtained, clustering into 12,240 OTUs with over 97% sequence similarity among sixteen samples. Four samples per group were used for this analysis. The rarefaction curves and species accumulation boxplot showed adequate sequencing depth for allthe samples (Figure 5A,B). The Venn diagram showed that 1228 OTUs were shared by all groups, and the number of unique OTUs in group LPC0, LPC0.1, LPC0.25, and LPC0.5 was 584, 906, 1290, and 1534, respectively (Figure 5C).

**Figure 6:**
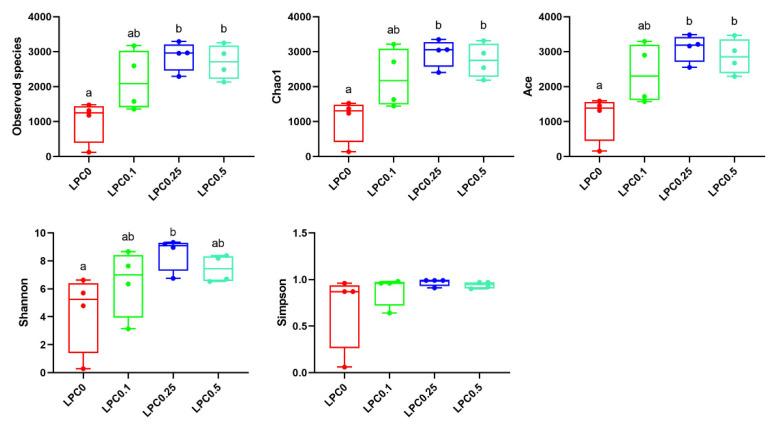
α diversity of the intestinal microbiota. Results were expressed as means ± S.E., and different superscript letters indicate significant differences (*p* < 0.05).

At the phylum level, Firmicutes and Proteobacteria were the predominant bacterial phyla, while the relative abundance of Bacteroidetes was next to them among all groups. At the genus level, the top 10 dominant genera included *Mycoplasma*, *Ralstonia*, *Bacteroides*, *Prevotella*, *Fusobacterium*, *Alteromonas*, *Escherichia-Shigella*, *Alistipes*, *Faecalibacterium*, and *Parvimonas* (Figure 5D,E). The ratios of Firmicutes to Bacteroidetes (F/B) in the LPC-supplemented groups were lower compared to the control group (*p* > 0.05) (Figure 5F). The results of the α-diversity analysis showed that the diets with 0.25% and 0.5% LPC significantly increased the bacteria richness (Observed species, Chao1, and Ace) (*p* < 0.05), while the diet with 0.1% LPC slightly increased these indices (*p* > 0.05). A significantly increased Shannon index was observed in the LPC0.25 group (*p* < 0.05) (Figure 6).

**Figure 7:**
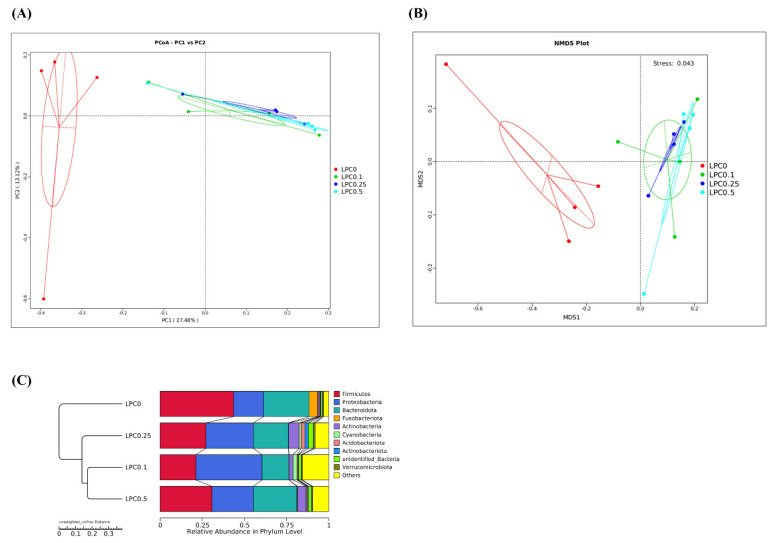
β diversity of the intestinal microbiota. (**A**) Principal Coordinate Analysis (PCoA) based on unweighted unifrac distances; (**B**) Non-metric Multidimensional Scaling (NMDS) based on unweighted unifrac distances; (**C**) Unweighted Pair-Group Method with Arithmetic mean (UPGMA)-clustering trees based on unweighted unifrac distances.

The results of the AMOVA test showed that the intestinal microbial community profile in the LPC-supplemented groups was significantly different from that in the LPC group (*p* = 0.026, 0.006, and 0.028, respectively) (Table S2). Similarly, the results of the PCoA plot, NMDS plot, and UPGMA showed that the intestinal bacteria compositions of the LPC-supplemented groups were distinctly separated from the LPC0 group (Figure 7).

**Figure 8:**
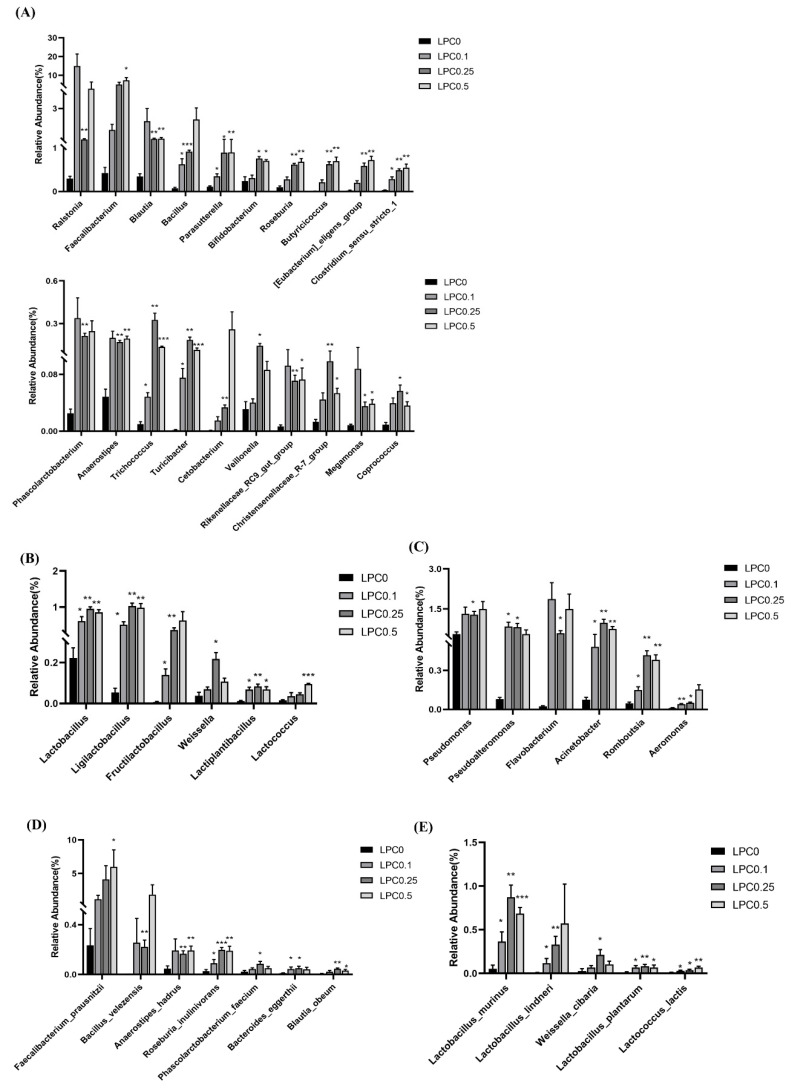
Metastat analysis of the intestinal microbiota communities among groups at different taxonomy. (**A**–**C**) Potential beneficial bacteria in intestinal microbiota at genus level. (**A**) Short-chain fatty acid producer, (**B**) Lactic acid producer, (**C**) Digestive enzyme-producing bacteria, (**D**,**E**) Potential beneficial bacteria in intestinal microbiota at species level. (**D**) Short-chain fatty acid producer (**E**) Lactic acid producer. * means significant difference between the LPC0 group and the LPC0.1, LPC0.25 or LPC0.5 group (* *p* < 0.05, ** *p* < 0.01, *** *p* < 0.001).

MetaStat analysis was conducted to compare the relative abundance of intestinal bacteria at genus and species levels among all groups. In the LPC-supplemented groups, the relative abundance of some potential beneficial bacteria, such as Faecalibacterium, *Pseudomonas*, *Blautia*, *Bacillus*, *Lactobacillus*, *Ligilactobacillus*, *Pseudoalteromonas*, *Parasutterella*, *Bifidobacterium*, etc., were obviously increased (*p* < 0.05) (Figure 8A–C). At the species level, diets with LPC supplementation significantly increased the relative abundance of *Faecalibacterium prausnitzii*, *Bacillus velezensis*, *Lactobacillus murinus*, *Lactobacillus lindneri*, *Anaerostipes hadrus*, *Roseburia inulinivorans*, *Weissella cibaria*, *Lactobacillus plantarum*, *Phascolarctobacterium faecium*, etc., (*p* < 0.05) (Figure 8D,E). The abundance of *Clostridium perfringens* was also significantly increased in the LPC-supplemented groups (*p* < 0.05) (Figure S2).

**Figure 9:**
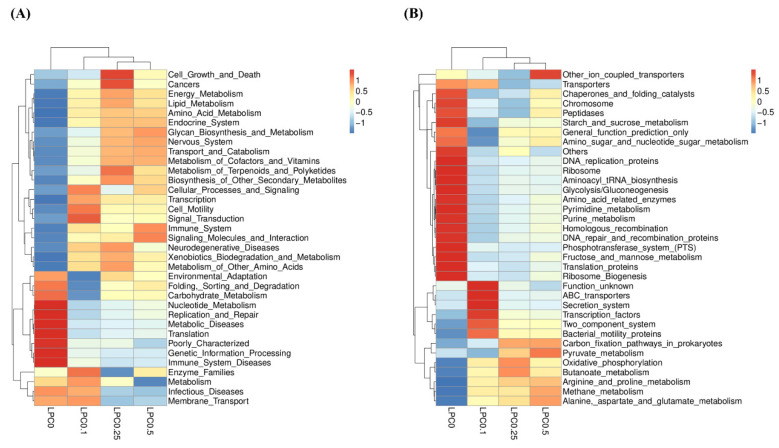
The functional profile predicted at level 2 (**A**) and level 3 (**B**) KEGGs Orthologs.

Based on PICRUSt-predicted KEGG orthologies (KOs), the heatmap showed that the communities in the LPC-supplemented groups were functionally different from those in the LPC0 group (Figure 9). At level 2, the relative abundance of the following gene functions, such as energy metabolism, lipid metabolism, amino acid metabolism, endocrine system, transcription, cell motility, signal transduction, immune system, and signaling molecules, and interactions were highly represented, while functions such as nucleotide metabolism, replication and repair, metabolic diseases, translation, and immune system diseases were relatively less abundant in the LPC-supplemented groups. At level 3, the genes involved in oxidative phosphorylation, butanoate metabolism, arginine and proline metabolism, methane metabolism, and alanine, aspartate and glutamate metabolism were relatively more abundant in the LPC-supplemented groups. However, compared with the LPC0 group, the relative abundance of DNA replication proteins, ribosome, aminoacyl tRNA biosynthesis, glycolysis/gluconeogenesis, amino acid-related enzymes, pyrimidine metabolism, and purine metabolism was visibly decreased in the LPC-supplemented groups.

### 3.6. Growth Performance

Fish grew normally in the present experimental conditions. The survival of fish in the LPC0 groups was lower than LPC-supplemented groups (*p* < 0.05). Compared to the LPC0 group, the weight gain in groups LPC0.1 and LPC0.25 was significantly higher (*p* < 0.05), with the highest value observed in the LPC0.1 group and an intermediate value observed in the LPC0.5 group. As the growth performance was not the focus of this study, the detailed growth data has been published elsewhere [28], and thus not described and discussed in the present study.

## 4. Discussion

According to the intestine histological results in the present study, intestinal inflammation was induced by high-lipid diets (LPC0), indicated by the inflammatory reactions and the villi atrophies. Lysophosphatidylcholine (LPC) was known to exert anti-inflammatory actions, suppressing leukocyte infiltration and pro-inflammatory cytokines [42]. Consistently, no inflammatory symptoms were observed in turbot fed diets with LPC supplementation in the present study. Meanwhile, a previous study in nursery pigs suggested that dietary LPC could increase the height of mucosal folds [43]. Similar evidence was provided by the current morphometric results: the height of mucosal folds (hMF), muscularis mucosa (hMM), and microvilli (hMV), and the perimeter ratio (PR) in the LPC0 group were relatively lower than those in the LPC-supplemented groups. It has been reported that the height of mucosal folds was determined by a balance between the rates of proliferation and the death of intestinal epithelial cells [44]. Therefore, improvements of intestinal morphology by dietary LPC might be closely associated with the inflammatory response in the intestinal mucosal barrier and the proliferation and apoptosis of intestinal epithelial cells.

A recent study in broilers has demonstrated that the Toll-like receptor (TLR) signaling pathway was one of the potential mechanisms involved in intestinal morphology improvement by dietary LPC [20]. However, to date, no study has investigated the effects of dietary LPC on the TLR signaling pathway in fish. In the present study, the diets with LPC supplementation significantly downregulated the gene expression of TLRs, including TLR3, TLR8, TLR9, and TLR22. Furthermore, both IRF-3 and MyD88 were evidently decreased. In consideration of these results, the TLR signaling pathway may play a role in the potential effects of LPC supplementation on intestinal physiology. Meanwhile, as known, high-lipid diet-induced intestinal damage was associated with the disruption of the intestinal mucosal barrier in both humans and fish [15,45]. Previous studies in Nile tilapia (*Oreochromis niloticus*) and rice field eel (*Monopterus albus*) have demonstrated that high-lipid diets can stimulate the TLR/NF-κB signaling pathway to regulate the expression of inflammatory cytokines [11,17]. In the present study, the significantly lower levels of signaling molecule NF-κB, as well as inflammatory cytokines (IL-1β and TNF-α), which were vital for the intestinal immune barrier, were found in the LPC-supplemented groups. A significantly decreased expression of IRF-3 was also observed in the LPC groups. In addition, a previous study in teleost fish found that the knockdown of IRF-3 inhibited the NF-κB signaling pathway [46]. Therefore, the intestinal immune barrier could be protected by dietary LPC via regulation of the TLR/NF-κB signaling pathway.

Meanwhile, another signaling molecule activated by TLRs could be mitogen-activated protein kinases (MAPKs), including ERK, JNK, and p38, which are important for regulating cell apoptosis. After being initiated by MAPKs, transcriptional factor AP-1 cascade is activated, which is responsible for the regulation of Bcl-2 family expression. It is generally considered that mitochondrial dysfunction, regulated by the Bcl-2 family of proteins (Bcl-2 and Bax), is a pivotal sign of cell apoptosis which can further activate the caspases such as Caspase-9 and Caspase-3 to execute cell apoptosis [47]. In turbot, it has been reported that intestinal epithelial cell apoptosis could be initiated by a high-lipid diet [14]. In the present study, the gene expression of signaling molecules JNK and AP-1 was evidently downregulated by dietary LPC, and the visibly decreased gene expression of apoptosis-related factors such as Bax, Caspase-9, and Caspase-3 was observed in the LPC groups. Furthermore, the gene expression of proliferative cell nuclear antigen (PCNA), the marker of cell proliferation, was upregulated by dietary LPC in this study. A previous study in vascular smooth muscle cells showed that the inhibition of p38 increased the gene expression of PCNA [48]. Therefore, the protection of the intestinal physical barrier by dietary LPC may be regulated by the TLR-mediate JNK and p38 signaling pathway.

Intestinal microbiota plays a fundamental role in the maintenance of intestinal homeostasis. The present study suggested that dietary LPC might have beneficial effects on the intestinal microbiota of turbot fed high-lipid diets. The results of α-diversity showed that diets with LPC supplementations, in particular the 0.25% group, increased the richness and diversity of intestinal microbiota. Meanwhile, the AMOVA test results showed that dietary LPC significantly altered the compositions of intestinal microbiota, and the same results were obtained according to the analysis of PCoA plot, NMDS plot, and UPGMA plot. In addition, phyla Proteobacteria, Firmicutes, and Bacteroidetes, which composed over 50% of phyla, were the predominant bacteria in the turbot intestinal mucosa across all groups. This was consistent with other studies in turbot [49,50]. Additionally, changes in the relative abundance of Firmicutes and Bacteroidetes were associated with host health. Numerous studies in both mammals and fish have reported that high-lipid diets caused a higher Firmicutes/Bacteroidetes (F/B) ratio, which was closely related to the imbalance of intestinal inflammation and other metabolic diseases. In this study, lower F/B ratios were found in the LPC-supplemented groups, suggesting that dietary LPC could mitigate the adverse impacts of high-lipid diets on intestinal microbiota. There were some differences in the top 10 bacteria at the genus level among groups. A higher proportion of *Mycoplamas*, *Prevotella*, *Fusobacterium*, and *Alistipes*, all of which were highly related to inflammation and other diseases [51,52,53,54], was found in the LPC0 group.

In order to further elucidate how intestinal microbiota was involved in the effects of LPC, the metastat analysis was used to deeply assess the alterations at genus and species levels. The present results showed that the proportions of short-chain fatty acids-producers (SCFAs-producers), lactic acid bacteria (LAB), and digestive enzyme-producing bacteria were much higher in the LPC-supplemented groups. SCFAs-produces *Faecalibacterium* (especially *F. prausnitzii*), an indicator of intestinal health, and *Bacillus* (especially *B. velezensis*), which has been evidenced to be able to ameliorate intestinal inflammation [55,56], had comparatively higher proportion in turbot diets with LPC. Meanwhile, a recent study in turbot showed that the dietary *Bacillus* species enhanced the abundance of *Ralstonia*, which was also significantly positively correlated with immune parameters such as AKP and ACP [57]. Interestingly, current results showed that a relatively higher abundance of *B. velezensis* and *Ralstonia* were found in the LPC-supplemented groups. Whether these bacteria were linked with each other or not needs future investigation. Additionally, previous studies have also reported that bacteria such as *Blautia*, *Parasutterella*, *Bifidobacterium*, *Roseburia*, and *Butyricicoccus* exerted anti-inflammatory effects by producing SCFAs, and some of them were closely associated with the inhibition of lipid accumulation [58,59]. Although LAB is not dominant in the normal intestinal microbiota of fish, some strains can colonize the intestine and then play important roles in the protection of host health [60,61]. A previous study has shown that the *Lactobacillus* population in broilers was increased by lysophospholipids (LPL) supplementation in the diet [23]. Similarly, in this study, the abundance of LAB belonging to *Lactobacillus*, *Ligilatobacillus* (especially *L. murinus*), *Fructilactobacillus* (especially *L.lindneri*), *Weissella* (especially *W. cibaria*), *Lactiplantibacillus* (especially *L. plantarum*), and *Lactococcus* (especially *L. lactis*) was enhanced in fish-fed diets with different levels of LPC supplementation. Some bacteria such as *F. prausnitzii*, *Anaerostipes*, *Veillonella*, and *Coprococcus* could utilize lactate and convert it largely to SCFAs, in particular butyrate, to avoid lactate accumulation in the fish intestine [62,63,64]. Furthermore, bacteria such as *Pseudomans*, *Pseudoalteromonas*, *Acinetobacter*, and *Aeromonas*, genera of Proteobacteria, which produce amylase, protease, lipase, or cellulase to improve the digestive processes of fish, had higher abundance in the LPC-supplemented groups. This helped to explain the relatively higher abundance of Proteobacteria in the LPC-supplemented groups [65]. The diets with 0.1–0.5% LPC also significantly enhanced the abundance of *Romboutsia*, which is characterized by producing bile salt hydrolase, synthesizing amino acids and vitamins, and utilizing simple carbohydrates [66]. The present results that higher proportions of digestive enzyme-producing bacteria were found in the LPC-supplemented groups were consistent with the previous results which showed that dietary LPC could significantly improve lipid digestion in turbot [28]. Interestingly, *Clostridium perfringens*, a type of diacylglycerol (DAG)-producing bacteria, was also evidently increased in the LPC groups. This might be associated with the higher concentration of DAG found in the turbot muscle [67,68]. However, there were few studies on DAG-producing bacteria to date. Therefore, to deeply investigate the involved mechanisms, further studies may correlate the alteration of some bacteria and changes in lipid profiles of the turbot muscle.

With regard to predictive function, the PICRUSt analysis showed that 0.1–0.5% LPC resulted in more microbial functional genes related to lipid metabolism and the immune system, and less genes related to metabolic diseases and immune system diseases, indicating that intestinal microbiota may play a vital role in the regulation of lipid metabolism and the improvement of immunity by dietary LPC. Butyrate, the preferred fuel of intestinal epithelial cells, can regulate the expression of cytokines or their receptors in the intestine, and enhance the intestinal barrier integrity [69]. The butanoate metabolism pathway participates in the butyrate synthesis route. In the present study, both the increased abundance of SCFAs-producers and the highly represented microbial genes involved in butanoate metabolism suggested that higher butyrate production may occur in the intestine of turbot supplemented with LPC. The results above indicate that 0.1%–0.5% LPC supplementation has positive effects on the composition and function of the intestinal microbiota in turbot fed high-lipid diets.

Acid phosphatase (ACP) and alkaline phosphatase (AKP) are not only important intestinal brush border enzymes, but also, pivotal components of natural defense, participating in immune regulation. Numerous evidence has shown that dietary probiotics could increase ACP and AKP activities. AKP is also important for the protection of the intestinal barrier through the maintenance of intestinal microbiota [7,8,57]. In the present study, activities of intestinal ACP and AKP were increased in the LPC-supplemented groups, and the highest values were shown in groups with 0.25% and 0.5%LPC, respectively. These results were in good agreement with the higher abundance of the immune system in the predictive function of intestinal microbiota.

The current results showed that dietary 0.1–0.5% LPC had beneficial effects on the intestinal health of turbot fed high-lipid diets. However, the previous study showed that only 0.1–0.25% LPC in the diet significantly improved the growth performance of turbot-fed high-lipid diets [28]. Therefore, the relevant mechanisms involved need more deeply investigation in the future.

## 5. Conclusions

Dietary LPC (0.1–0.5%) had protective effects on the intestinal health of turbot fed high-lipid diets. Diet with LPC supplementation regulated the intestinal immune and physical barriers. The TLR-mediated NF-κB pathway and TLR-mediated JNK and p38 signaling pathways might play important roles in the regulations mentioned above, respectively. The composition and function of turbot intestinal microbiota were also significantly altered by dietary LPC, which may contribute to the regulation of intestinal health by LPC. However, further investigation is needed to elucidate whether the TLR signaling pathway is suppressed directly by LPC or indirectly via some bacteria and their metabolites.

## Figures and Tables

**Table 1:** Formulation and proximate composition of the experimental diets (% dry matter).

Ingredients	Group			
	LPC0	LPC0.1	LPC0.25	LPC0.5
Fish meal	40	40	40	40
Soy protein concentrate	10	10	10	10
Soybean meal	8	8	8	8
Wheat meal	21.68	21.68	21.68	21.68
Brewer’s yeast	5	5	5	5
Mineral premix ^a^	0.5	0.5	0.5	0.5
Vitamin premix ^a^	1	1	1	1
Monocalcium phosphate	1	1	1	1
L-ascorbyl-2-polyphosphate	0.2	0.2	0.2	0.2
Choline chloride	0.2	0.2	0.2	0.2
Betaine	0.3	0.3	0.3	0.3
Ethoxyquin	0.02	0.02	0.02	0.02
Calcium propionic acid	0.05	0.05	0.05	0.05
Fumaric acid	0.05	0.05	0.05	0.05
Fish oil	5.5	5.5	5.5	5.5
Soybean oil	5.5	5.5	5.5	5.5
Soya lecithin	1	0.9	0.75	0.5
Lysophosphatidylcholine (LPC)	0	0.1	0.25	0.5
Proximate Compositions				
Moisture	6.4	6.67	6.68	6.8
Crude protein	44.13	44.01	44.02	43.72
Crude lipid	14.86	15.86	15.31	16.34
Ash	8.05	8.01	8	8.08

^a^ Vitamin premix and mineral premix, designed for marine fish, were purchased from Qingdao Master Biotech Co., Ltd., Qingdao, China.

## Data Availability

Raw data supporting the conclusions of this manuscript will be made available by the authors, without undue reservation, to any qualified researcher.

## Supplementary Materials

The following supporting information can be downloaded at: https://www.mdpi.com/article/10.3390/nu14204398/s1, Figure S1: Intestinal length index (ILI) and intestinal somatic index (ISI) of turbot; Figure S2: Effect of dietary LPC on the abundance of *Clostridium perfringens* in distal intestine of turbot; Table S1: Primers used for quantitative real-time PCR analysis; Table S2: AMOVA test of distal intestine.
