# Supplementary material for: Effects of Lysophosphatidylcholine on Intestinal Health of Turbot Fed High-Lipid Diets
Source: Nutrients. 2022 Oct 20;14(20):4398. doi: 10.3390/nu14204398 (PMC9611283; doi:10.3390/nu14204398)
Supplement: Supplementary file 1 [file nutrients-14-04398-s001.zip › nutrients-1959335-supplementary.pdf]

Effects of lysophosphatidylcholine on intestinal health of turbot fed high-lipid diets

Sihui Li [1], Xing Luo [2], Zhangbin Liao [2], Mengqing Liang [2,3], Houguo Xu [2,3,*], Kangsen Mai [1,3] and Yanjiao Zhang [1,3,*]

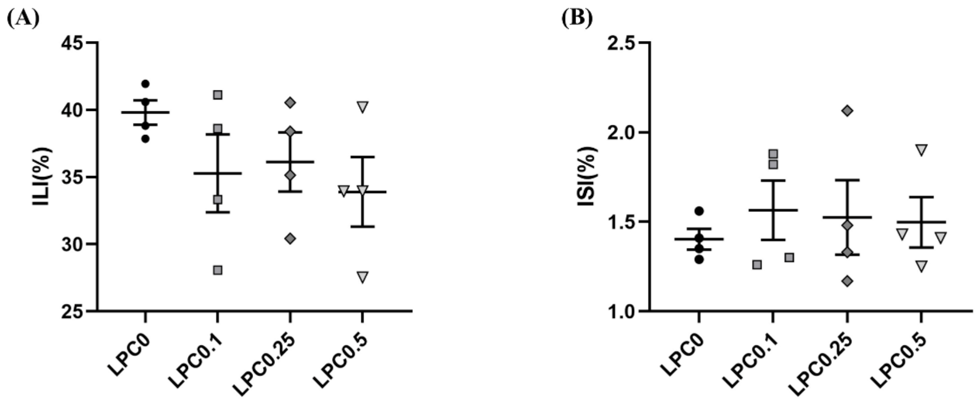

Figure S1. Intestinal length index (ILI) and intestinal somatic index (ISI) of turbot.

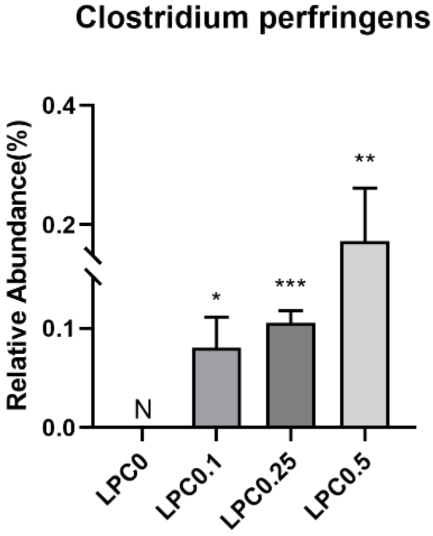

Figure S2. Effect of dietary LPC on the abundance of *Clostridium perfringens* in distal intestine of turbot. *means significant difference between the LPC0 group and the LPC0.1, LPC0.25 or LPC0.5 group as evaluated by T-test ($^{*}P < 0.05$, $^{**}P < 0.01$, $^{***}P < 0.001$).

Table S1. Primers used for quantitative real-time PCR analysis.

Target Genes	Sequence (5'-3')	Accession umber
AP-1	F: CAGCTGCGGCTTGAAGTTTT<br>R: GTTCTACGACGAAGCCGTGA	(Zhao et al.,2019)
Bax	F: AGCATCTTTGCTGACGGGAT<br>R: GCGCTCTCTGATGACCTGAA	MN782169
Bcl-2	F: TTCCTCAACTCTCAAAGCACAATTC<br>R: ATTACACTCGCTCGCCATTCC	MN782168
Caspase-3	F: TTCTGCCATTGTCTCTGTGC<br>R: GCCCTGCAACATAAAGCAAC	JU391554.1
Caspase-9	F: CCCAGGACATGATCGACGAG<br>R: ACAATGGGAAGGCTCGACTG	XM_035644988.1
ERK	F: TCAACCACATACTGGGCATCC<br>R: TCGAGTCGGCCTTGAAGAA	(Zhao et al.,2019)
IFN-γ	F: GCTTTCCCGATCATCTTCTG<br>R: GGTTTCCCAGATTCCCATTC	DQ400686.1
IL-1β	F: CGCTTCCCCAACTGGTACAT<br>R: ACCTTCCACTTTGGGTCGTC	XM_035640817
IRF3	F: TGGGAGAAGAAACTCATCACA<br>R: ACATCTCCATCATCTCTTCCA	HQ424131.1
JNK	F: CAGCCAACAGACCAGACA<br>R: TGAAGCCCAGTAACATCG	(Zhao et al.,2019)
MyD88	F: CCCAATGGTAGCCCTGAGAT<br>R: CATCTCGGTCGAACACACAC	KP985236.1
NF-κB	F: ACACTGCTGAGCTGAAGATC<br>R: CTCTGAGCCCATCAGGGTC	MF370855
PCNA	F: CTCGTACCGCTGCGACAG<br>R: CAGAGGGCATCTTCACCAC	EU711051.1
p38	F: GACGAACCCTGTTTCCTGGT<br>R: TCGGCTGCTGTTATTCGCTT	(Zhao et al.,2019)
TGF-β1	F: CTGCAGGACTGGCTCAAAGG<br>R: CATGGTCAGGATGTATGGTGGT	KU238187.1
TLR2	F: AGGAGCCAAGGGAGACCGAT<br>R: GGCGCTCATGATGTTGTCC	KU746963.1
TLR3	F: TGGTGTCGTCGATTCAAAGC<br>R: CCAATCCAACTACTCCCCACG	KX216854.1
TLR5a	F: AGTCTCTTTGGTCTCAGGGC<br>R: TTTGGGTAAGACATCGGGCT	KX525706
TLR5b	F: AACAACTTCCTAGCCTCCCC<br>R: CATGTGAAATCCTCCGCTGG	KX525707
TLR8	F: ACAGATCCTTGAACTCCCCG	KX708702.1

	R: TCCAATCCCTCTCCTCCAGA	
TLR9	F: AAGGCTCTGAGGGGAAAGAC<br>R: TTCTTCACAGAGCTGAGGGG	KU746969.1
TLR21	F: CAGCTGTCATCCTATCACCG<br>R: TTGTCATTGCCCTGCGTAG	KU746965.1
TLR22	F: ACAGAGACTTCGAGCCAGGT<br>R: CTTGTTCGGCAGTTTCCTCA	KJ606344.1
TNF-α	F: GGACAGGGCTGGTACAACAC<br>R: TTCAATTAGTGCCACGACAAAGAG	AJ295836.2
β-actin	F: CGTGCGTGACATCAAGGAG<br>R: AGGAAGGAAGGCTGGAAGAG	AY008305.1

Abbreviations: AP-1 activator protein-1; Bax B-cell lymphoma associated X; Bcl-2 B-cell lymphoma-2; Caspase cysteine-aspartic proteas; ERK extracellular regulated kinase; IFN-γ interferon-γ; IL interleukin; IRF interferon regulatory factor; JNK c-Jun N-terminal kinase; MyD88 myeloid differentiation factor 88; NF-κB nuclear transcription factor-kappa B; PCNA proliferating cell nuclear antigen; p38 p38 mitogen-activated protein kinase; TGF-β1 transforming growth factor-β1; TLR toll like receptor; TNF-α tumor necrosis factor-α.

Table S2. AMOVA test of distal intestine (*n*=4).

Groups	LPC0-LPC0.1	LPC0-LPC0.25	LPC0-LPC0.5
P-value	0.026*	0.006*	0.028*

* means significant difference between the LPC0 group and LPC-supplemented groups ($P < 0.05$).
